# Seeded Synthesis of AlPO_4_-5 Membrane in Diluted Mother Liquor

**DOI:** 10.3390/membranes12121284

**Published:** 2022-12-19

**Authors:** Jing Wang, Chao Ji

**Affiliations:** Cener Tech Tianjin Chemical Research and Design Institute Co., Ltd., Tianjin 300131, China

**Keywords:** AlPO_4_-5 membrane, hydrothermal, pure component permeance, mother liquor

## Abstract

AlPO_4_-5 with an AFI topology membrane on an a-alumina substrate has been fabricated continuously, without defects, and with high intergrowth. By using traditional hydrothermal methods in diluted mother liquor, an AlPO_4_-5 membrane has been produced by a simple second growth synthesis. Scanning electron microscopy (SEM) revealed no defects in the prepared supporting film, and X-ray (XRD) diffraction confirmed the layer of molecular sieve AlPO_4_-5 on the porous support of α-alumina. In this study, various synthesis parameters were systematically examined. Based on H_2_, He, N_2_, CO_2_, and SF_6_ permeance results, the supported membranes display Knudsen diffusion behavior, and the membrane’s pervaporation properties of organic compounds (n-hexane, o-xylene, and TIPB) show minimized defects, verifying their high quality.

## 1. Introduction

Flanigen and colleagues reported the first AlPOn molecular sieves in 1982 [1]. AFI-structured molecular sieves are an important member of the aluminum phosphate molecular sieve family, which also includes metal-substituted derivatives with 12-ring channels in one dimension parallel to the [002] direction, such as AlPO_4_-5, SAPO-5, and MeAPO-5 (0.73 nm in diameter) [2]. AFI zeolite membrane synthesis with c-axis orientation is a hot spot in the field of zeolite molecular sieve membrane research due to its unique pore structure [3,4,5]. Molecular sieve AlPO_4_-5 (AFI) is a representative phosphate molecular sieve, due to its valuable structural properties, including catalysis, catalytic films, chemical sensors, and highly selective membrane separation [6,7,8,9].

Zeolite membranes have been studied extensively around the world as an appealing tool for process development for the separation of liquid and gas components. The separation process’s goals include dehydration, organic–organic separation, gas separation, and so on. Growing c-oriented AlPO_4_-5 membranes from irregular seeds was a recent study on this topic [3]. A seed layer of oriented zeolite crystals on a porous substrate coated with a thin layer of silica was used to grow continuous, well-grown zeolite membranes using secondary growth. Significant progress has also been made in the direct in situ crystallization of oriented AlPO_4_-5 zeolite membranes on a carrier [10,11,12,13,14,15,16,17,18]. The conventional hydrothermal synthesis of zeolite membranes is not only difficult to synthesize but also wastes a huge amount of raw materials, and the commercial application of zeolite membranes is limited by high unit cost, unsatisfactory separation performance (low permeability and selectivity), irreproducible synthesis, and poor ductility of zeolite membranes. In particular, the unit cost of zeolite membranes, estimated to be about USD 2000/m^2^, is significantly higher than that of its direct competitor, polymer membranes, and must be addressed first [19,20,21].

Danni M. discusses the ball milling process used to produce ultra-small SAPO-34 seed particles. Smaller seeds stimulate secondary nucleation, which reduces film thickness even more than tailored nano-SAPO-34 seeds, resulting in thinner membranes with improved CO_2_ permeability [22]. In the absence of fluoride medium, high-silicon CHA-type aluminosilicates (Si/Al molar ratio > 100) were produced through a hydrothermal process, with a seed-assisted aging treatment playing a significant role in crystallization [23]. The influence of different factors on the synthesis of all-silica CHA in a diluted mother liquor with a H_2_O/SiO_2_ ratio of 30 was investigated by Kong et al. The effective synthesis of all-silica CHA zeolite in diluted mother liquor simplifies not only its production, but also its uses as solvents and membranes [24]. Senlin Y. described a novel approach for synthesis of aluminum-deficient mother solutions that used a self-terminating mechanism to ensure minimum film development. The material efficiency is great, and the film thickness is easy to adjust [25].

As a molecular sieve with a one-dimension channel, the AlPO_4_-5 molecular sieve must not only modify the thickness of the molecular sieve membrane, but also the orientation of the molecular sieve to change the permeability of the molecular sieve membrane. By etching micron-sized AlPO_4_-5 molecular sieves into fragments and coating them on a carrier as crystals, we were able to synthesize zeolite membranes in diluted mother liquor using triethylamine (TEA) as a template in this study. An investigation of the effects of different H_2_O/ML (mother liquor) ratios on the synthesis of AlPO_4_-5 membranes was conducted. The molecular sieve membranes grown on porous alumina can be used for both continuous and reactive separation processes. We can synthesize AlPO_4_-5 membranes using conventional hydrothermal synthesis, which simplifies the process and allows us to control the membrane size and morphology of AlPO_4_-5 crystals. Furthermore, the cost is lower, and the process is simpler. It would be ideal to develop a simple (low-cost) method for producing AlPO_4_-5 zeolite membranes.

XRD and SEM were used to characterize the samples. Pure gas component permeance is used primarily to determine whether a membrane is of high quality.

## 2. Experimental Details

### 2.1. Chemicals

The gel composition directly affects the composition and properties of the product. Triethylamine (TEA, 99%, Tianjin Shengmiao Technology, Tianjin, China) was used as a template, and silica sol (SiO_2_, 30 wt.%, Tianjin Shengmiao Technology), pseudo-boehmite (AlOOH·nH_2_O, 70 wt.%, Shanxi Juhua Co., LTD., Taiyuan, Shanxi, China), and analytically pure phosphoric acid (H_3_PO_4_, 85 wt.%, Tianjin Shengmiao Technology, Tianjin, China) as the raw materials.

For our experiments, we used homemade substrates, with α-Al_2_O_3_ disks that were 20 mm in diameter, 2 mm thick, and had an average pore diameter of 0.20 μm. A smooth surface was obtained by polishing only one side of the substrate with 800 and 1200 mesh sandpaper. In the ultrasonic bath, the substrates were cleaned for 2 min with deionized (DI) water. A DI water wash was followed by a 2 h immersion in ethanol. They were then dried at 100 °C for 5 h before being calcined at 650 °C for 3 h.

### 2.2. Synthesis of AlPO_4_-5 Microcrystals

Typically, TEA is dispersed in deionized water in order to synthesize AlPO_4_-5 microcrystals. The gel used for hydrothermal synthesis had the following composition: 1.0Al_2_O_3_:1.3P_2_O_5_:1.2TEA:56H_2_O. After adding the pseudo-boehmite to the TEA solution, the mixture was vigorously stirred for one hour. A 20 min stirring process was then performed, with the H_3_PO_4_ solution added to the above mixture. After that, 10 minutes of vigorous stirring was performed with the SiO_2_ sol–gel added to the above liquid. The stirring rate was set to 500 rpm. To finish the process, the slurry was placed in a 100 mL pressure container with Teflon-lined stainless steel for ten hours at 150 °C. Flowing water cooled the autoclave, and the solid zeolites were collected, cleaned, and dried after vacuum filtration with deionized water.

### 2.3. Synthesis of AlPO_4_-5 Membrane

**Figure 1:**
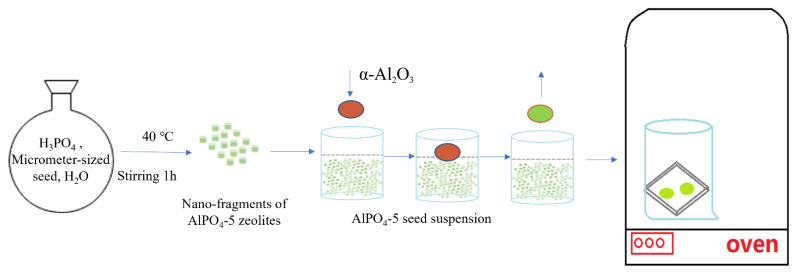
Schematic diagram of dip-coating method of AlPO_4_-5 seed layer on α-Al_2_O_3_ support.

First, the membrane was made by etching the micron-sized AlPO_4_-5 crystals into fragments and dispersing them in H_3_PO_4_ solution. The small fragments of AlPO_4_-5 seeds were used as primary crystal nuclei in the crystals [26]. After choosing the dip coating, we immediately seeded the layer for second growth. Based on the results of this study, a dip-coating method is proposed for the preparation of the AFI seed layer on α-Al_2_O_3_ support, as illustrated in Figure 1.

Membrane1 (M1) was prepared using micron-sized crystals of AlPO_4_-5 as the seed from synthesized solutions of the molar composition: 1.0 Al_2_O_3_:1.3P_2_O_5_:1. 2TEA:400H_2_O. Using micron-sized crystals of AlPO_4_-5 as the seed, membrane2 (M2) was prepared in a mixture of H_2_O and mother liquor with a 3-to-1 ratio. For comparison, the hydrothermal synthesis was repeated, and membrane3 (M3) was obtained by etching AlPO_4_-5 ultra-small fragments as the seed from the molar composition of the synthetic solutions: 1.0 Al_2_O_3_:1.3P_2_O_5_:1. 2TEA:400H_2_O. At the same time, membrane4 (M4) with etched AlPO_4_-5 ultra-small fragments as the seed was synthesized with a H_2_O/ML (mother liquor) ratio of 3 in a diluted mother liquor.

To increase substrate coverage and produce well-intergrown membranes, experiments needed to go through a longer reaction time. A 100 mL Teflon autoclave was filled with the precursor gel of ca. 30–40 mL, in which the α-alumina substrate was placed face down on the bottom. We sealed the Teflon autoclave and placed it in a furnace which was already heated to 423 K for 24 h (M1, M3), 10 h (M2, M4). After the synthesis, we cooled it to room temperature and removed the sample, washed it with deionized water, and dried it under the conditions of 313 K and 40% humidity for two days. The membrane was calcined at 573 K for 3 h, and then 873 K for 3 h, to remove the template. At a rate of 0.5 K/min, the membrane was heated and cooled.

### 2.4. Characterization

An X-ray diffractometer of the Bruker-AXS D8 (Bruker, Bremen, Germany) was used to measure AlPO_4_-5 particle structure and crystallinity. A S-4800 SEM (Hitachi, Tokyo, Japan) was used to image morphology and particle size.

### 2.5. Permeation Test

During this study, pore and membrane structure, molecular size, and adsorption affinity were discussed in relation to permeance selectivity of single gases in AlPO_4_-5 membranes. The permeances of H_2_, He, CO_2_, N_2_, CO, and SF_6_ were determined through the zeolite membranes. We built our own apparatus for testing gas permeation. The feed pressure of all the gases above differed from that of permeation side. The feed pressure was maintained at 20 MPa. The atmospheric pressure was set on the permeation side. The permeation rates of gases were measured at 298 K.

## 3. Results and Discussion

Micron seeds cannot be used as beneficial seeds if they cannot be broken into fragments by either acids or bases. According to the results of this study, a dip-coating method can be used to prepare AlPO_4_-5 seeds layers, as illustrated in Figure 1. In the synthesized gel, SAPO-5 seeds are first etched into fragments and evenly scattered under vigorous stirring. The primary crystal nuclei of SAPO-5 seeds are nano-sized fragments. Then, using the dip-coating process, an AlPO_4_-5 seed layer was produced on α-Al_2_O_3_ support.

**Figure 2:**
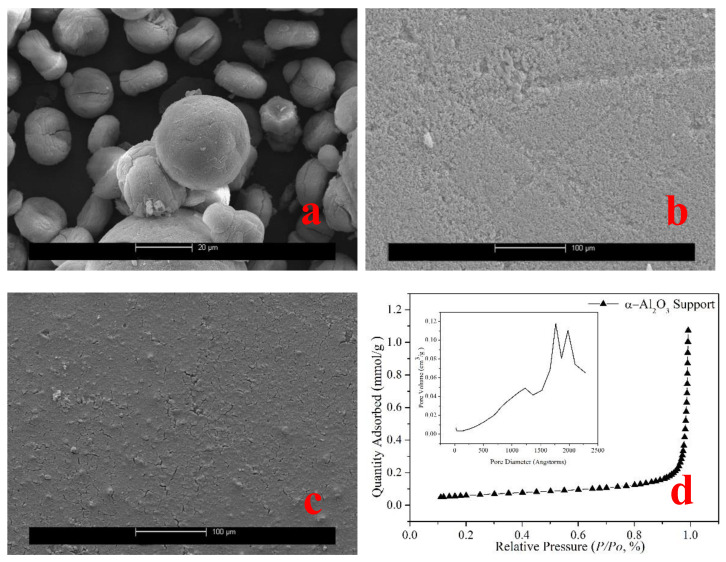
(**a**) SEM picture of the AlPO_4_-5 seeds powder, (**b**,**c**) SEM image of the α-Al_2_O_3_ substrate and the AlPO_4_-5 seeds deposited on α-Al_2_O_3_ substrate. (**d**) Nitrogen adsorption isotherm of α-Al_2_O_3_ support. Inset is the pore size distribution obtained from the adsorption branch by the BJH method.

The powder comprises huge spherical particles of 10~20 μm, as seen in Figure 2a. Figure 2b shows that the particle size on the carrier’s surface is not uniform, that there are gaps between the particles, and that the surface is extremely rough. Figure 2c depicts the shape of the deposited seeds on α-Al_2_O_3_ substrates. SEM scans reveal that the seeds are well covered on the surfaces. Figure 2d shows the nitrogen adsorption isotherms of α-Al_2_O_3_ support. According to the BET and BJH models, the material’s surface area is 5.3 m^2^/g, and the pore distribution is mostly focused in the 180–220 nm region. Thus, the α-Al_2_O_3_ support can be considered a macroporous material.

**Figure 3:**
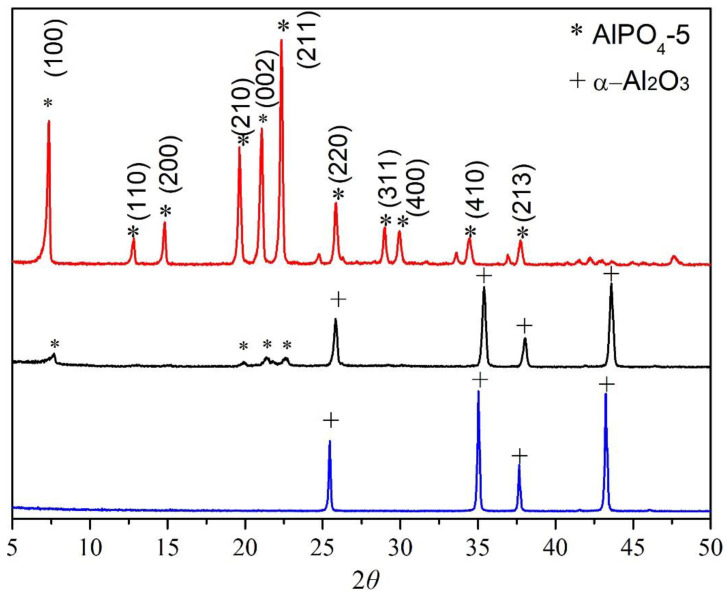
XRD images of AlPO_4_-5 particles being used prepare seeds, α-Al_2_O_3_ substrate, and AlPO_4_-5 seeds deposited on α-Al_2_O_3_ substrate.

The plus signs in Figure 3 reflect peaks arising from the α-Al_2_O_3_ substrate, whereas the asterisks represent peaks originating from the AlPO_4_-5 layer. The usual peaks of the AFI framework structure may be easily observed in the XRD pattern, suggesting AlPO_4_-5 crystals on the α-Al_2_O_3_ support.

**Figure 4:**
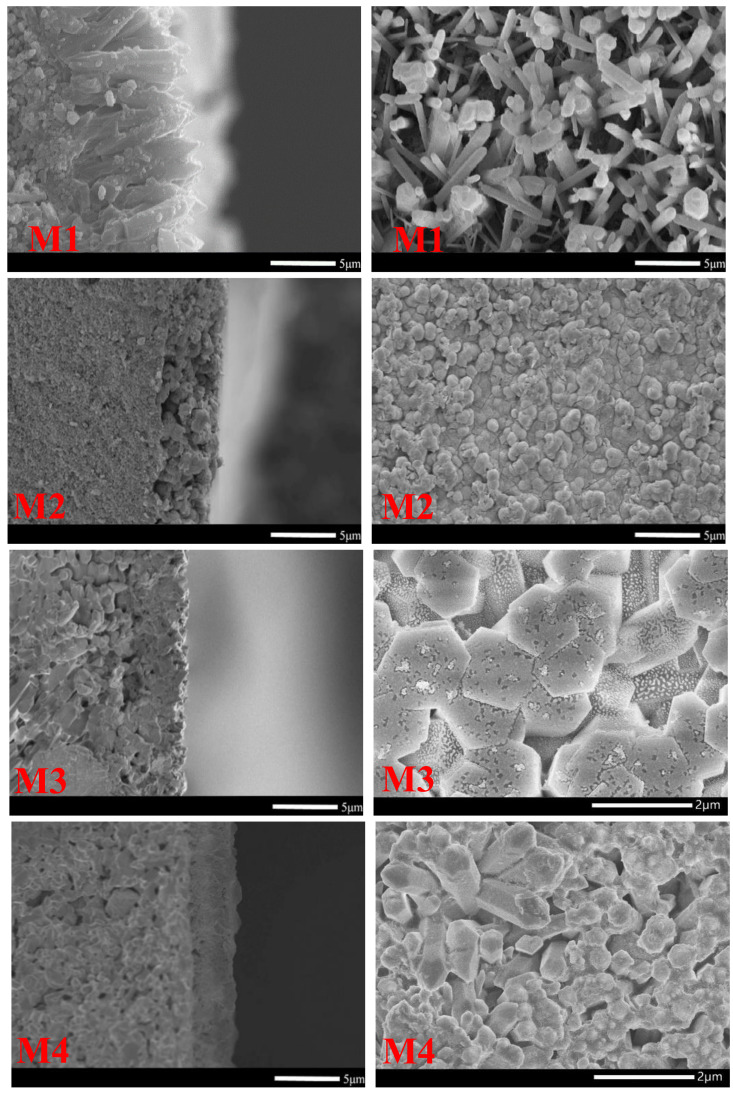
SEM top view (**right**) and cross-section (**left**) of AlPO_4_-5 membrane second growth on α-Al_2_O_3_ support.

**Figure 5:**
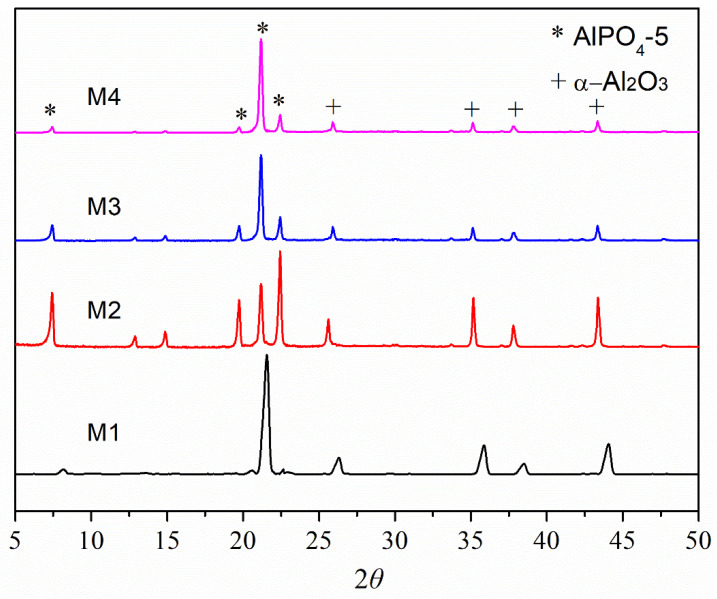
XRD pictures of AlPO_4_-5 crystals pre-oriented with the c-axis perpendicular to the substrate following secondary growth in a favorable c-plane growth environment. The plus signs indicate peaks that originate from the α-Al_2_O_3_ substrate, whereas the asterisks indicate peaks that originate from the AlPO_4_-5 layer.

In Figure 4, SEM images show all the synthesized AlPO_4_-5 membranes after being dip coated and dried in the oven for the second growth. Experiments using varying molar compositions of the precursor mixture revealed that the mother liquor concentration had a significant impact on crystal shape. Without the addition of mother liquor, a badly intergrown yet pre-oriented membrane was formed after secondary development (Figure 4—M1). The compact membrane was obtained, but the crystal shape changed from rode to ball by adding mother liquor (Figure 4—M2) and the membranes were too thick and random. The kind of sol with mother liquor with the ratio of 2H_2_O/mother liquor created powdered coin AlPO_4_-5 crystals, which were then employed for secondary growth on a-Al_2_O_3_ substrates (Figure 4—M3). At the 3H_2_O/mother liquor ratio, the AlPO_4_-5 grains formed a membrane with an average thickness of 3 μm, according to SEM analysis (Figure 4—M4) (perpendicular to a substrate). The crystal orientation was affected by the combination of broken seed pieces and mother liquid. When the concentration is high, the synthesis of molecular sieves tends to be spherical. The molecular sieve increases toward the c-axis direction when the mother liquid is gradually diluted. When the concentration is low, the hexagonal crystal is formed. Secondary growth was used to create the pre-oriented and random membranes, based on XRD analysis. As shown in Figure 5, the XRD characteristic peaks of zeolite are quite strong, belonging to the normal AFI peak without an impurity phase and a strong alumina peak, suggesting that the support surface is coated by a thin AlPO_4_-5 film layer. Peaks of 7.5°, 20°, 20.97°, and 22.5° were found, showing that the molecular sieve membranes were placed randomly on the support surface (Figure 5—M2 and M3). In Figure 5, the peak at 2θ of 20.97° corresponds to the AlPO_4_-5 framework’s greatly enhanced (002) reflection, suggesting that the channels are perpendicular to the substrate (Figure 5—M1 and M4). In this paper, we offer a unique approach to producing oriented molecular sieve membranes that may be used for separating and catalyzing. Comparing SEM and XRD patterns, we can see that when concentration increases, AlPO_4_-5 molecular sieves prefer to develop spherically, resulting in a disordered state. When the concentration is moderately lowered, AlPO_4_-5 grows along the epitaxial growth, and when the concentration is further reduced, the molecular sieve grows along the c-axis.

**Figure 6:**
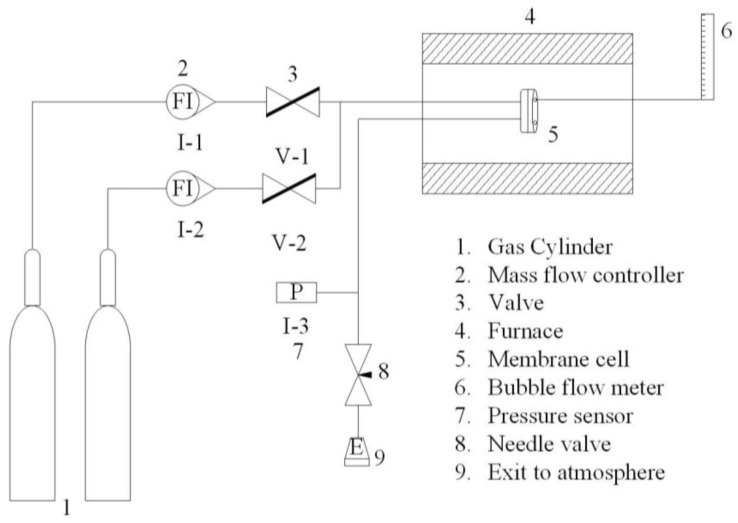
The steady-state permeation setup is depicted schematically.

**Figure 7:**
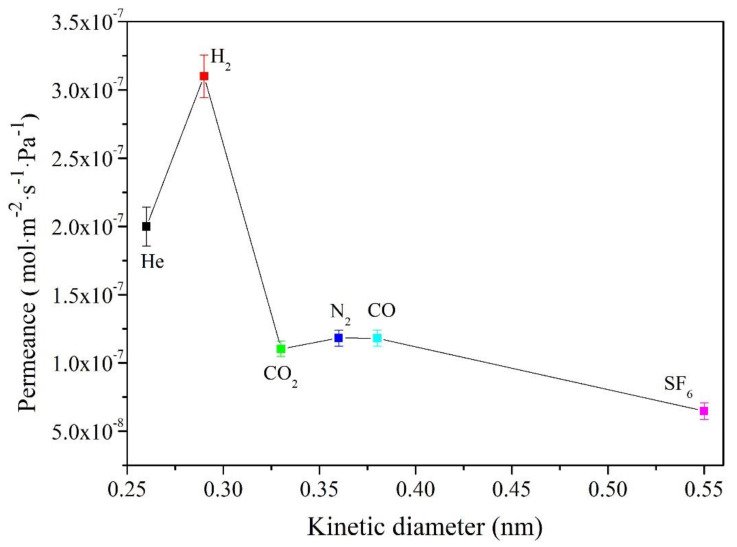
Permeation flux of single gas He, H_2_, CO_2_, N_2_, CO, and SF_6_ on AlPO_4_-5 membrane at 298 K. The standard deviations of the permeate flux data are presented with error bars of 5%.

The steady-state permeation setup (Figure 6) for the gas permeation tests were homemade. Generally, the gas permeation fluxes decrease as the molecular size increases. However, a conclusion was drawn through the results of the permeation flux of single gas in the membrane (Figure 7). Both H_2_ and CO have strong permeation fluxes compared with the He and CO_2_. Therefore, CO is presumed to have weak absorbability. In addition, the diffusion of single gas molecules exhibits that it is the Knudsen diffusion mechanism.

The permeance of n-hexane, o-xylene, and TIPB across the AlPO_4_-5 membrane developed in this work is shown in Table 1. Because the dynamic molecular diameters of n-hexane (0.43 nm) and o-xylene (0.68 nm) are both smaller than that of AlPO_4_-5 (0.73 nm), they may flow smoothly through the AlPO_4_-5 membrane at close to 10^−5^ mol·m^−2^·s^−1^·Pa^−1^. The molecular TIPB (0.85 nm) is slightly larger than the theoretical aperture of AlPO_4_-5 (0.73 nm). As a result, its penetration flow on the AlPO_4_-5 membrane is significantly lower than that of n-hexane and o-xylene. The flow rate of AlPO_4_-5 membrane penetration to TIPB is 0.5 × 10^−11^ mol·m^−2^·s^−1^·Pa^−1^. The AlPO_4_-5 crystal membrane has less non-AFI channels and defective pores, and its quality is excellent.

## 4. Conclusions

AlPO_4_-5 membrane has been grown directly on the porous α-alumina by utilizing traditional hydrothermal methods in a diluted mother liquor. As has been shown, by using this less complex and cheaper method, we created a continuous and densely intergrown thinner AlPO_4_-5 membrane. According to scanning electron microscopy (SEM), the prepared supported membrane is defect free. In addition, the creation of an AlPO_4_-5 molecular sieve membrane layer on an alumina porous substrate is confirmed by X-ray diffraction (XRD) research. Results of the permeance tests conducted on the supported membranes (H_2_, He, N_2_, CO_2_, SF_6_) indicate Knudsen diffusion behavior, and the membrane’s pervaporation properties of organic compounds (n-hexane, o-xylene and TIPB) show minimized defects, which confirms good membrane quality.

## Figures and Tables

**Table 1:** List of organic molecules for testing the quality of AlPO-5 membranes and pervaporation flux measured.

Chemicals	Molecule Size (Å)	Molecule Weight (g/mol)	Permeance (mol·m^−2^·s^−1^·Pa^−1^)
n-hexane	4.3	86.18	1.2 × 10^−5^
o-xylene	6.8	106.17	0.7 × 10^−6^
TIPB	8.9	204.35	0.5 × 10^−11^

## Data Availability

All the data generated or analyzed within the present investigation are included in this manuscript.
